# Hypogammaglobulinemia with Facial Edema

**DOI:** 10.1371/journal.pmed.0030475

**Published:** 2006-12-26

**Authors:** Adina Kay Knight, Lloyd Mayer, Andrew G Franks, Charlotte Cunningham-Rundles

## Abstract

Knight and colleagues discuss the diagnosis and management of a 35-year-old man with a past history of recurrent cellulitis and otitis media and a two-year history of facial swelling.

## DESCRIPTION of CASE

A 35-year-old man came to the office with right-sided facial swelling, which he had noted over the last two years. The swelling was worse in the mornings and decreased through the day; however there were no symptom-free days. He had not experienced fevers, chills, or changes in his vision. He had experienced multiple episodes of lower extremity cellulitis, left more frequent than right leg, beginning in childhood. Although most instances were treated with oral antibiotics, he required hospitalization for intravenous antibiotics at least four times. Previous evaluation had confirmed lymphedema by radionucleotide clearance. In addition, he had symptoms of allergic rhinitis and two episodes of otitis media as an adult, but he had no history of pneumonia or other significant respiratory or gastrointestinal infections.

On physical examination he had no evidence of wasting or malnutrition. He had normal tonsils and no cervical lymphadenopathy; there was mild right facial swelling with induration and trace erythema from the eye to mid-cheek. The right facial skin was slightly warmer than the left, but it was non-tender to palpation. Tympanic membranes and chest examination were normal. His legs were moderately swollen, left greater than right, with a “woody” or indurated texture to the left leg; there were no rashes or other skin lesions. He had no hepatosplenomegaly.

## What Investigations Are Indicated in This Patient?

Frequent infections raise the possibility of immunodeficiency. A targeted immunologic evaluation should be guided by the clinical symptoms as well as the relative frequency of known immunodeficiencies.

Immune defects in the humoral system are most common and screening can be performed with tests for serum immunoglobulin levels and titers of specific antibody. A suggestion of immunoglobulin deficiency arises if there is a low total protein on standard chemistry panels, as the immunoglobulins make up a considerable portion of serum proteins. Clinical symptoms of immunoglobulin deficiency include increased frequency or severity of sino-pulmonary and other bacterial infections.

Cellular immune deficiencies are suggested by opportunistic and viral infections. An initial step in the evaluation of these is a complete blood count (a low lymphocyte number can be missed if only total white cells are counted) followed by a lymphocyte panel enumerating CD4 and CD8 T cells as well as B cells and natural killer cells. It is important to obtain the lymphocyte evaluation with a standard complete blood count to allow for the calculation of absolute numbers of cells and not just percentages, since normal relative percentages may be preserved despite very low cell numbers. More subtle defects in T cell function may be investigated by examining lymphocyte proliferative responses to mitogens and soluble antigens.

Other rarer immune function defects, such as neutropenia or neutrophil dysfunction (e.g., chronic granulomatous disease leading to recurrent skin or organ abscesses), complement defects (systemic bacterial infections or meningitis), and IL-12 and interferon gamma axis dysfunction (mycobacterial infections), are less likely in this adult patient without clinical history or infections characteristic of these conditions.

Laboratory data included low serum immunoglobulin G (IgG) 268 mg/dl (694–1618) and immunoglobulin M (IgM) 18 mg/dl (48–271,) but normal serum immunoglobulin A (IgA) 119 mg/dl. Electrolytes, kidney, and liver function tests were normal. Serum calcium was 7.8 mg/dl (normal 8.5–10.4), total protein was 4.8 g/dl (normal 6.0–8.3), albumin was 2.9 g/dl (normal 3.7–5.1), and calculated globulin was 1.9 g/dl (normal 2.2–4.2). The urinalysis was normal with no protein detected. Further studies revealed normal white blood cell count (7.8 × 10^3^/μl), hemoglobin, hematocrit, platelet count, and normal numbers of neutrophils, monocytes, eosinophils, and basophils. He had reduced numbers of lymphocytes 0.5 × 10^3^/μl (normal 1.0–4.5 × 10^3^/μl), consisting of reduced T cells 293 (750–2500/cu mm), CD4 T cells 238 (480–1700/cu mm), and CD8 T cells 40 (180–1000/cu mm), an increased CD4/CD8 ratio 6.10 (1.00–3.00), slightly low numbers of natural killer cells, 90 (135–525/cu mm), and normal numbers of B cells 85 (75–375/cu mm).

## What Is the Differential Diagnosis?

Decreased immunoglobulin levels can result from reduced production or increased loss (Table 1). Primary causes of hypogammaglobulinemia are the genetic B or T cell defects. Secondary causes of reduced immunoglobulin production can be malignancy (lymphoma, thymoma, leukemia, multiple myeloma), selected medications (carbazepine [1], oxcarbazepine [2], immunosuppressive agents [3], and others), or infections such as Epstein-Barr virus, perinatally acquired HIV, or starvation. Increased nonselective loss of immunoglobulin can occur in rare states of high catabolism or with protein loss through protein-losing enteropathy (Table 2), drainage of ascites, or chylothorax (lymph fluid in the pleural space).

**Table 1:**
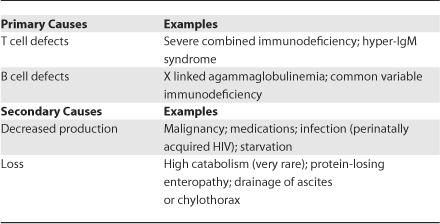
Causes of Hypogammaglobulinemia

**Table 2:**
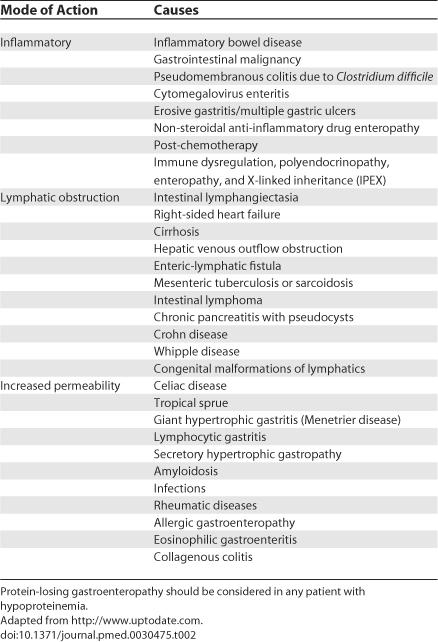
Causes of Protein-Losing Enteropathy

Lymphedema may predispose to recurrent cellulitis in affected limbs, however, given the significant hypogammaglobulinemia found here, an intrinsic defect in the immune system leading to an inability to eliminate infections should be investigated.

## What Additional Laboratory Data or Investigations Would Be Helpful in Making a Diagnosis in This Patient?

The ability to make specific antibody after vaccination challenge can assist in differentiating between decreased production and increased loss of immunoglobulins. Defects in specific IgG antibody production are characteristic of primary immune defects such as X-linked agammaglobulinemia, hyper-IgM syndrome(s), and common variable immunodeficiency (CVID). Patients with hypogammaglobulinemia due to protein loss would be expected to respond normally to vaccinations in generating plasma B cells producing specific IgG antibody, though there may be lower serum immunoglobulin levels due to the general loss of proteins including IgG antibodies.

This man was first assumed to have a primary immune deficiency such as CVID, a syndrome most often associated with sino-pulmonary infections. The diagnosis of CVID is made by documenting decreased serum IgG, and IgA and/or IgM and poor specific antibody production [4]. Significant improvement in infections is expected when IgG is replaced by immunoglobulin therapy [5]. CVID was considered a likely diagnosis, since it is often made during the second or third decade of life, however it is usually associated with a history of recurrent upper respiratory infections [6], not noted here.

However, other genetic immune defects and secondary causes, such as drug-induced, malignancy-associated hypogammaglobulinemia, or immunoglobulin loss, must be excluded. X-linked agammaglobulinemia and hyper-IgM were considered improbable due to the patient's age, mild clinical history, normal numbers of B cells, normal serum IgA, and normal tonsillar tissue.

Specific IgG antibody titers to varicella, rubella, and tetanus were found to be in a range considered to be protective, though the patient lacked sufficient antibody to measles, mumps, and 12 serotypes of pneumococci. After vaccination with measles, mumps, and rubella (MMR) and pneumococcal vaccine, he developed protective specific antibody titers to measles and mumps and nine out of 14 pneumococcal serotypes. The preserved specific antibody production in response to pneumococcal, measles, and mumps vaccination, with the protective titers of antibody to varicella, rubella, and tetanus demonstrated normal B cell function, and excluded CVID.

## How Did We Investigate the Possibility of a Protein-Losing Enteropathy?

Normal B cell function, low albumin, and the intermittent history of significant lymphedema suggested the possibility of protein loss as the explanation for the hypogammaglobulinemia [7]. Normal urinalysis and renal functions eliminated nephrotic loss, which would have been an extremely unusual etiology for this degree of hypogammaglobulinemia.

To evaluate gastrointestinal protein loss, stool alpha-1 antitrypsin was determined and found to be elevated at 625 mg/dl (normal <55 mg/dl). This result suggested the presence of a protein-losing enteropathy such as intestinal lymphangiectasia. Intestinal lymphangiectasia may also be associated with the loss of T cells through the lymphatic channels. Although we did not evaluate this in our patient, in general naïve T cells are preferentially lost over memory T cells due to their trafficking patterns though the lymphatic system [8,9]. However, the remaining T cells are generally able to prevent opportunistic infections. Intestinal lymphangiectasia was thus consistent with the patient's reduced numbers of T cells and low levels of serum immunoglobulins.

## What Was the Original Diagnosis?

Based on the presence of excess alpha-1 antitrypsin in the stool, the patient was diagnosed with protein-losing enteropathy. Conditions leading to protein-losing enteropathy can be congenital or acquired. Our patient has had symptoms suggestive of lymphatic dilatation since childhood and congenital lymphangiectasia was considered as a leading diagnosis. The most likely type of congenital lymphangiectasia would be hereditary lymphedema type I (Milroy disease) (Table 3). Hereditary lymphedema type I is the result of a defect in the *FLT4* gene, encoding vascular endothelial growth factor receptor-3 [10]. This is an autosomal dominant mutation with variable penetrance and expression in affected members of a family. Although our patient has no known affected family members, relatively mild disease may have been overlooked. However, the degree of facial swelling our patient experienced is not generally seen in this condition.

**Table 3:**
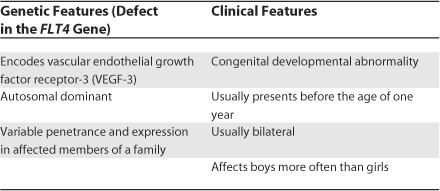
Features of Milroy Disease or Hereditary Lymphedema Type I

Targeted evaluation for secondary causes of intestinal lymphangiectasia was conducted. Cardiac history and echocardiography were unremarkable in our patient, excluding cardiac disease as a secondary cause of his protein-losing enteropathy. Upper and lower endoscopies by his local gastroenterologist did not reveal any visible anomalies indicative of Crohn disease, ulcerative colitis, or other lesions, suggesting that our patient's protein loss was likely due to either a diffuse process or one occurring in the non-visualized small bowel.

## How Is Lymphangiectasia Treated?

Treatment for intestinal lymphangiectasia includes treatment of the underlying pathology if a secondary cause is present (Table 4). Our patient's history is not consistent with any of the secondary causes of protein-losing enteropathy and he has chronic lymphedema of the face and extremities as well. Congenital intestinal lymphangiectasia can be surgically resected if there is an isolated segment of affected bowel. Most often there is diffuse intestinal involvement precluding resection. Other modalities have been used, including an extremely low fat diet to reduce the lymph flow to the intestinal mucosa, dietary medium chain triglycerides, or medications including heparin and octreotide [11–14]. However, our patient's normal intestinal biopsy (no dilated lymphatics characteristic of lymphangiectasia) suggested these treatments were unlikely to be effective.

**Table 4:**
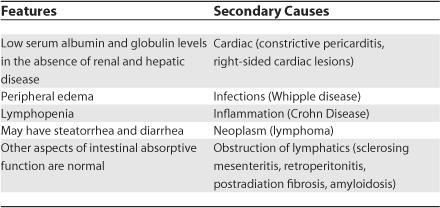
Features and Secondary Causes of Intestinal Lymphangiectasia [15–19]

## What Was the Final Diagnosis?

**Figure 1:**
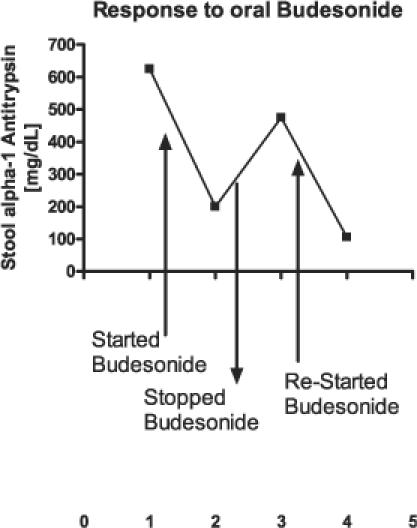
Response to Oral Budesonide Stool protein loss, as measured by alpha-1 antitrypsin, decreased with oral locally active rapidly metabolized steroids and increased when the steroids were stopped, suggesting a steroid responsive gastrointestinal inflammation as the underlying cause of the protein loss (including loss of immunoglobulins).

Since his history and biopsy findings were not consistent with congenital or acquired lymphangiectasia, an inflammatory etiology was considered; thus a diagnostic trial of topical oral steroids (budesonide) was given. After several weeks of use, the patient experienced a significant decrease in facial and extremity lymphedema. Laboratory evaluation also revealed a decrease in stool protein loss with decreased stool alpha-1 antitrypsin levels (Figure 1) suggesting an inflammatory component to his protein-losing enteropathy. Based upon the response to steroids, an inflammatory gastrointestinal process was considered to be the underlying cause of his protein-losing enteropathy and subsequent hypogammaglobulinemia, but no specific etiology was identified.

**Learning Points**

- Hypogammaglobulinemia can result from primary or secondary causes.
- Secondary causes of hypogammaglobulinemia should be sought before diagnosing a patient with primary hypogammaglobulinemia.
- Protein-losing enteropathy is a cause of secondary hypogammaglobulinemia and is associated with a low albumin as well as nonspecific serum protein loss.
- T cells can also be lost in protein-losing enteropathies.
- Not all hypogammaglobulinemia requires intravenous immunoglobulin replacement.

With good specific antibody production to vaccination challenge and no history of serious infections, replacement immunoglobulin therapy with intravenous immunoglobulin is not indicated. Continued close surveillance and a low threshold for the use of antibiotics would be prudent as he is at higher risk of recurrent cellulitis due to his lymphedema.

## Conclusion

The differential diagnosis of hypogammaglobulinemia includes evaluation for both primary and secondary causes, since the treatment for these conditions is different. Protein-losing conditions can lead to hypogammaglobulinemia and loss of T cells, but immune function is generally preserved and immunoglobulin replacement therapy is not usually required.

## Abbreviations

IgA: immunoglobulin A
IgG: immunoglobulin G
IgM: immunoglobulin M
CVID: common variable immunodeficiency
